# Navigation of Multiple Disk-Shaped Robots with Independent Goals within Obstacle-Cluttered Environments

**DOI:** 10.3390/s23010221

**Published:** 2022-12-25

**Authors:** Panagiotis Vlantis, Charalampos P. Bechlioulis, Kostas J. Kyriakopoulos

**Affiliations:** 1 Department of Electrical and Computer Engineering, University of Patras, University Campus, 26504 Rion, Greece; 2 School of Mechanical Engineering, National Technical University of Athens, 15780 Athens, Greece

**Keywords:** multi-robot navigation, motion planning, cell decomposition

## Abstract

In this work, we propose a hybrid control scheme to address the navigation problem for a team of disk-shaped robotic platforms operating within an obstacle-cluttered planar workspace. Given an initial and a desired configuration of the system, we devise a hierarchical cell decomposition methodology which is able to determine which regions of the configuration space need to be further subdivided at each iteration, thus avoiding redundant cell expansions. Furthermore, given a sequence of free configuration space cells with an arbitrary connectedness and shape, we employ harmonic transformations and harmonic potential fields to accomplish safe transitions between adjacent cells, thus ensuring almost-global convergence to the desired configuration. Finally, we present the comparative simulation results that demonstrate the efficacy of the proposed control scheme and its superiority in terms of complexity while yielding a satisfactory performance without incorporating optimization in the selection of the paths.

## 1. Introduction

The autonomous operation of robotic platforms inside cluttered environments constitutes an actively studied research topic, with autonomous navigation undeniably being a fundamental aspect of it. Additionally, as the tasks that robots are entrusted with grow in complexity, the employment of multi-robot systems, which generally exhibit a higher robustness and versatility than their single-robot alternatives, progressively increases. Thus, the multi-robot navigation problems to be addressed become more challenging day by day, raising the need for more efficient path and motion planning schemes.

Several methodologies can be found in the literature for coordinating the motion of two or more robots such that a desired configuration is reached under standard collision avoidance specifications. Combinatorial and algebraic approaches, such as the ones presented in [1,2], were among the earliest to be considered. However, despite their elegance, their complexity renders them impractical for addressing cases with non-trivial sizes of robotic teams. On the other side, probabilistic sampling methods, such as Rapidly Exploring Random Trees [3] and Probabilistic Roadmaps [4], constitute a popular solution employed in the recent literature due to their simplicity and their ability to efficiently handle large configuration spaces while being subjected to a variety of constraints. Nevertheless, these methodologies are generally having a hard time addressing problems with constricted configuration spaces (e.g., workspaces densely occupied by obstacles or narrow corridors). To alleviate these issues, various attempts were made. In [5], a novel sensory steering algorithm was designed which used the local Voronoi decomposition of the workspace to significantly improve the path-planning performance of sampling-based algorithms near difficult regions, such as narrow passages. In [6,7], the authors proposed a scheme that samples entire manifolds instead of isolated configurations, which are, in turn, used for approximating the configuration space’s connectivity graph, thus allowing the planner to perform significantly better even in tight workspaces. Alternatively, when a common graph representation of the workspace is shared among the agents, efficient methodologies for coordinating their transitions were proposed in [8,9,10,11,12]. On the other hand, a methodology was presented in [13,14], which addresses cases where the motion of each robot is restricted to a distinct graph by building a composite roadmap (i.e., the Cartesian product of the individual graphs). Furthermore, more efficient extensions of this approach, which work on implicitly defined composite roadmaps and, potentially, lower-dimensional configuration spaces, can be found in [15,16,17,18]. The approximate cell decomposition [19] and Slice Projection [20,21,22] constitute alternative methodologies which were successfully employed for tackling robot navigation problems with complex configuration spaces [23,24,25] and generally exhibit fast exploration capabilities when coupled with hierarchical adaptive subdivision schemes guided by suitable heuristics. For more details on the related literature, the reader may refer to the following recent comprehensive review paper [26].

**Figure 1:**
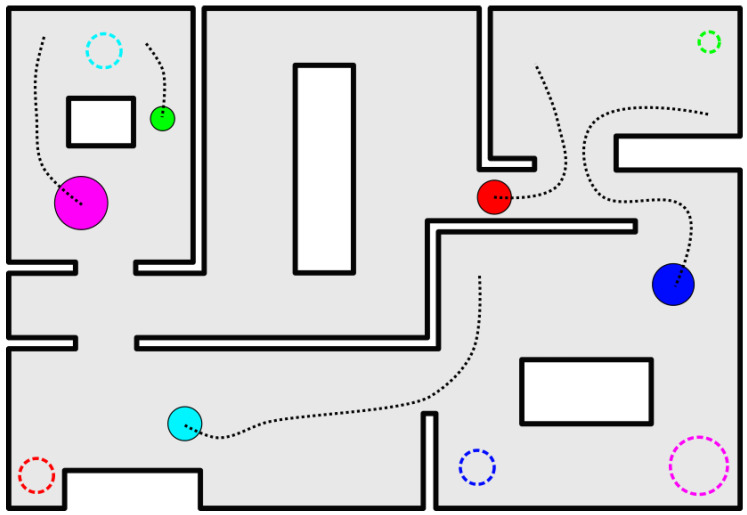
Multiple robots (solid colored disks) navigating to their corresponding goal configurations (dashed circles).

In this work, we address the navigation problem for a team of disk-shaped robots that operate within an arbitrary, obstacle-cluttered, planar and connected workspace (see Figure 1). Given an initial and desired configuration for the robotic team, we design a high-level hierarchical cell decomposition planner which is tasked with the exploration of the system’s configuration space to discover a sequence of cells that the robots can safely traverse toward the desired configurations. One of the strong points of the proposed algorithm is the use of a suitable labeling mechanism for selecting the regions of the configuration space to be subdivided at each iteration. Particularly, by computing an over- and an under-approximation of each robot’s footprint, our algorithm can determine which cells may contain feasible configurations of the system while automatically discarding the cells that are determined to contain none. Finally, having obtained a sequence of traversable cells, we equip each robot with decentralized low-level control laws based on harmonic maps and adaptive harmonic potential fields, originally introduced in [27], which guarantee the safe and almost-global convergence to the goal. It should be noted that the high-level planner produces a series of cells through which the multi-robot system has to go in order to attain the final desired configuration. The aforementioned low-level controller, which was extracted by our previous work [27], simply guarantees a safe transition between successive cells. Thus, any other motion planning algorithm would also suffice.

The contribution of this work is the adaptive subdivision algorithm for obtaining a sequence of traversable cells that connect the given initial and final configurations or determining that no solution exists. Unlike standard grid/cell-based methods, the proposed algorithm subdivides the cells adaptively, requiring no selection of some arbitrary resolution by the user. Moreover, unlike probabilistic sampling methods, the proposed method does not generate paths of configuration but rather sequences of safe sets, allowing the user to make use of a larger set of controllers in addition to standard trajectory tracking schemes. Additionally and contrary to the probabilistic sampling methods, which are inherently incapable of determining the infeasibility of a given problem (such algorithms require the user to specify an arbitrary upper bound of samples after which the algorithms abort) as a direct consequence of their probabilistic completeness nature, our algorithm partitions the configuration space into cells (dense subsets) of the configuration space and is capable of determining when no admissible path exists in finite time (see [19]).

The outline of this article is as follows. At the end of this section, we define some preliminary notions and the notation used throughout this work. In Section 2, we formulate the problem addressed in this work. In Section 3, we elaborate on the proposed planner’s design as well as the velocity control scheme employed for safely executing the computed plan. Finally, the simulation results verifying the efficacy of the proposed control scheme are presented in Section 4.

*Notation:* Throughout this work, we shall use $I_{N}≜\left\{1,2,\ldots ,N\right\}$ (resp., $I_{N}^{★}≜\left\{0\right\}\cup I_{N}$) to denote the set consisting of all natural numbers up to *N*, starting from 1 (resp., 0). Additionally, given sets *A* and *B*, we use ∂A, $\mathrm{int}\left(A\right)$ and $\mathrm{cl}\left(A\right)$ to denote the boundary, interior and closure of *A*, respectively, and A∖B to denote the complement of *B* with respect to *A*.

## 2. Problem Formulation

We consider a team of $N_{R}$ robots operating within a compact planar workspace $W\subseteq R^{2}$ occupied by a set of $N_{o}$ disjoint, fixed inner obstacles $O_{i},i\in I_{N_{o}}$. We assume that each robot *i* has a disk-shaped body $R_{i}\subset R^{2}$ with radius $r_{i}>0$. Let $F_{w}$ and $F_{i},i\in I_{N_{R}}$ be the coordinate frames arbitrarily embedded in W and $R_{i},i\in I_{N_{R}}$, respectively. We shall refer to the origin of each $F_{i},i\in I_{N_{R}}$ as the reference point of the corresponding robot. Moreover, without loss of generality, we assume that the reference point of each robot coincides with the center of its body. Let $p_{i}≜{\left[x_{i},y_{i}\right]}^{T}\in R^{2}$ denote the relative position of *i*-th robot’s reference point with respect to the workspace’s coordinate frame $F_{w}$, and let $R_{i}(p)$ denote its footprint, i.e., the space occupied by robot *i* when placed at position *p*. Throughout this work, we shall use $C\subseteq R^{2N_{R}}$ to denote the robotic system’s configuration space and $P≜{\left[p_{1}^{T},p_{2}^{T},\ldots ,p_{N_{R}}^{T}\right]}^{T}\in C$ to denote the stacked vector of robot positions. For the sake of brevity, we shall also use P[i] to denote the *i*-th component of *P*, i.e., $P\left[i\right]=p_{i}$. Let $W^{o}$ denote the complement of W, i.e., $W^{o}≜R^{2}\setminus W$. We also define a configuration *P* as feasible iff the following conditions hold:

(1) $$\begin{array}{cc} R_{i}(P[i])\cap R_{j}(P[j])=\emptyset ,\; & \;\;\;\forall i\neq j\in I_{N_{R}}\\ R_{i}(P[i])\cap W^{o}=\emptyset ,\; & \;\;\;\forall i\in I_{N_{R}} \end{array}$$

and we shall use  $C^{f}\subset C$ to denote the set of all feasible configurations of the robotic system, whereas its complement $C^{o}≜C\setminus C^{f}$ corresponds to the set all infeasible configurations. Finally, we assume that the motion of each robot *i* obeys the single-integrator kinematic model:

(2) $${\dot{p}}_{i}=u_{i},\;i\in I_{N_{R}}$$

where $u_{i}$ denotes the control input.

**Problem:** *Let $P_{\mathrm{init}}$ and $P_{\mathrm{des}}$ be two given feasible configurations of the multi-robot system that belong to the same segment of  $C^{f}$. Our goal is to design a control scheme that drives any robot i, initialized at $p_{\mathrm{init},i}=P_{\mathrm{init}}\left[i\right]$, to the specified desired position $p_{\mathrm{des},i}=P_{\mathrm{des}}\left[i\right]$, while avoiding inter-robot and robot–workspace collisions, i.e., $P\left(t\right)\in C^{f}$ for all t≥0.*

## 3. Control Design

To address the aforementioned problem, first, we employ a hierarchical cell decomposition scheme for partitioning the configuration space of the multi-robot system C into cells, as described in Section 3.1. Then, we design a high-level planner, in Section 3.2, which recursively expands the aforementioned structure until a sequence of adjacent cells connecting $P_{\mathrm{init}}$ and $P_{\mathrm{des}}$ is found. Finally, the low-level control scheme that ensures safe transition between cells until the goal configuration is reached is presented in Section 3.3.

### 3.1. Configuration Space Decomposition

In this subsection, we present the hierarchical cell decomposition scheme that will be employed in our approach. We begin with disregarding inter-robot collisions and considering the configuration space of each individual robot. Particularly, the configuration space of robot *i*, denoted herein by $A_{i}\left(W\right)$, corresponds to the largest subset of W where the reference point of robot *i* can be placed such that $R_{i}(p_{i})\cap W^{o}=\emptyset$, for all $p_{i}\in A_{i}\left(W\right)$. Moreover, given a subset Z of W, we shall use $A_{i}\left(Z\right)$ to denote the set of feasible positions of robot *i* which belong to Z, i.e.,

(3) $$A_{i}\left(Z\right)≜\left\{\;p\;|\;p\in Z\;\mathrm{and}\;R_{i}(p)\cap W^{o}=\emptyset \;\right\},\;\forall i\in I_{N_{R}}.$$

In addition to $A_{i}\left(\cdot \right)$, which corresponds to the set of feasible positions of robot *i*, we also consider two estimations of the area that is potentially occupied by $R_{i}$ when $p_{i}$ is restricted in a subset Z of W. Particularly, given a robot $i\in I_{N_{R}}$ and a set $Z\subseteq A_{i}\left(W\right)$, let $\bar{R_{i}}(Z)$ and $\underline{R_{i}}(Z)$ be an over-approximation and under-approximation, respectively, of the footprint of $R_{i}$ when robot *i* is swept over Z such that:

(4) $$\begin{array}{cc} \bar{R_{i}}(Z)\; & ⊇\overset{}{\underset{p\in Z}{⋃}}R_{i}(p)\\ \underline{R_{i}}(Z)\; & \subseteq \overset{}{\underset{p\in Z}{⋂}}R_{i}(p) \end{array}$$

and

(5) $$\begin{array}{c} \bar{R_{i}}(Z)\subseteq \bar{R_{i}}(Z\prime ),\;\;\;\forall Z\subseteq Z^{\prime }\\ \underline{R_{i}}(Z)⊇\underline{R_{i}}(Z\prime ),\;\;\;\forall Z\subseteq Z^{\prime }. \end{array}$$

**Figure 2:**
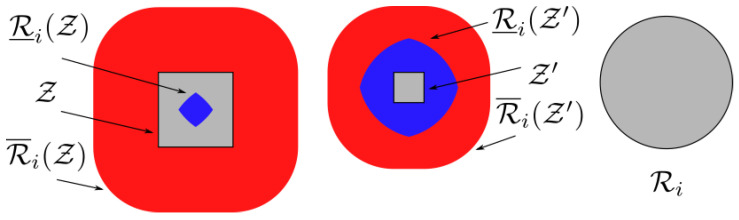
Over-approximation $R_{i}\left(Z\right)$ (resp., $R_{i}\left(Z^{\prime }\right)$) and under-approximation $R_{i}\left(Z\right)$ (resp., $R_{i}\left(Z^{\prime }\right)$) of the footprint of robot *i* when swept over $Z\subset R^{2}$ (resp., Z).

An example of such approximations can be seen in Figure 2. (for disk-shaped robots, a valid over-approximation $\bar{R_{i}}(Z)$ can be computed by offsetting Z by $r_{i}$, whereas $\underline{R_{i}}(Z)$ can be calculated by $\cap_{p\in \partial Z}R_{i}(p)$).

We now consider a set $S\subseteq R^{2}$ that has the form $\left[x_{1},x_{2}\right]\times \left[y_{1},y_{2}\right]$. We shall refer to such a set as a simple slice of $R^{2}$. Given a simple slice S and a robot $i\in I_{N_{R}}$, we will use $W_{S}^{i}≜A_{i}\left(W\right)\cap S$ to denote the set of feasible positions of robot *i* (neglecting inter-robot collisions) that are contained in S. A set $S=\left\{\;S_{i}\;|\;i\in I_{N_{S}}\;\right\}$ of $N_{S}$ simple slices shall be called a cover of W iff

(6) $$\begin{array}{c} W=\underset{j\in I_{N_{S}}}{⋃}S_{j}\cap W. \end{array}$$

**Figure 3:**
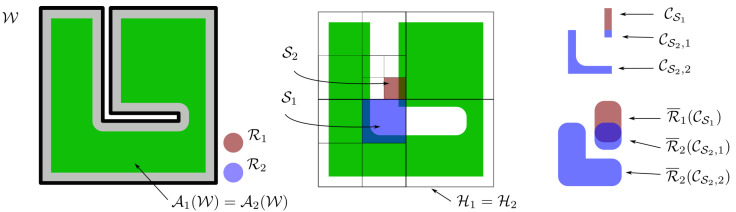
Example of a hierarchical configuration space decomposition for a system of two identical robots. The green area corresponds to the configuration space $A_{i}\left(W\right)$ of each robot. For the sake of simplicity, we assume $H_{1}=H_{2}$. The workspace slice $S_{1}$ corresponding to robot 1 consists of a single simple cell ($C_{S_{1}}$) whereas slice $S_{2}$ corresponding to robot 2 consists of two cells ($C_{S_{2},1}$, $C_{S_{2},2}$). The compound cell $\left(C_{S_{1}},C_{S_{2},1}\right)$ is labeled as mixed because $R_{1}$ and $R_{2}$ may intersect when $p_{1}\in C_{S_{1}}$ and $p_{2}\in C_{S_{2},2}$ because $\bar{R_{1}}\left(C_{S_{1}}\right)\cap \bar{R_{2}}\left(C_{S_{2},1}\right)\neq \emptyset$, whereas $\left(C_{S_{1}},C_{S_{2},2}\right)$ is marked as admissible.

We note that a cover S partitions $A_{i}\left(W\right)$ into a set of regions $W_{S}^{i},\;S\in S$ each of which consists of zero or more individually connected but pairwise disjoint subsets $C_{S,j}^{i},\;j\in I_{N_{L}\left(W_{S}^{i}\right)}$, which shall be referred to as workspace (or simple) cells. A cover $\hat{S}=\left\{\;\left(i,S_{i}\right)\;|\;i\in I_{N_{R}}\;\right\}$ of the configuration space C is, respectively, defined by assigning a cover to each robot. Accordingly, a configuration space (or compound) slice $\hat{S}$ is defined as $\hat{S}=\left\{\;\left(i,S_{i}\right)\;|\;i\in I_{N_{R}}\;\right\}$, where $S_{i},\;i\in I_{N_{R}}$ is a set of simple slices. Likewise, a configuration space cover $\hat{S}=\left\{\;\left(i,S_{i}\right)\;|\;i\in I_{N_{R}}\;\right\}$ induces a partitioning of C into regions ${\hat{W}}_{\hat{S}}≜W_{S_{1}}^{1}\times W_{S_{2}}^{2}\times \ldots \times W_{S_{N_{R}}}^{N_{R}}$, where $\hat{S}=\left\{\;\left(i,S_{i}\right)\;|\;i\in I_{N_{R}}\;\right\}$ is an element of $\hat{S}$. We note that each of these regions may consist of zero or more individually connected but pairwise disjoint subsets ${\hat{C}}_{\hat{S},i}^{},\;i\in I_{N_{L}\left(C_{\hat{S}}\right)}$, which shall be referred to herein as configuration space (or compound) cells. Given the compound cell ${\hat{C}}_{\hat{S},i}^{}$, we will use ${\hat{C}}_{\hat{S},i}^{\left[j\right]}$ to denote its *j*-th component, i.e., ${\hat{C}}_{\hat{S},i}^{\left[j\right]}≜C_{S_{j},i}^{j}$, for all $j\in I_{N_{R}}$. We remark that, unlike hierarchical decomposition schemes commonly encountered in the literature, which use cells of simple geometries (e.g., hypercubes or hyperrectangles), the configuration space cells considered in this work have, in general, arbitrary geometries because their components do not possess a pre-specified shape (see Figure 3). Although this choice renders navigation within a cell $\hat{C}$ more complicated, it generally results in coarser partitions because because each component ${\hat{C}}^{\left[i\right]}$ of $\hat{C}$ belongs in $A_{i}\left(W\right)$ by construction; thus, the subdivision scheme has to accommodate only for potential inter-robot collisions.

Regarding now the transition between configuration space cells, we introduce some required notions of connectedness. We begin with considering two distinct simple slices $S_{i}$ and $S_{j}$ which shall be called adjacent iff their intersection $S_{i}\cap S_{j}$ is not empty. Moreover, let $C_{S_{m},i}^{}$ and $C_{S_{n},j}^{}$ be two distinct workspace cells. We define these simple cells as adjacent iff $C_{S_{m},i}^{}\cap C_{S_{n},j}^{}\neq \emptyset$. Apparently, $C_{S_{m},i}^{}$ and $C_{S_{n},j}^{}$ being adjacent implies that $S_{m}$ and $S_{n}$ are also adjacent. The aforementioned definitions can be naturally extended to compound slices and cells, as well. Particularly, two compound slices ${\hat{S}}_{m}=\left\{\;\left(i,S_{m,i}\right)\;|\;i\in I_{N_{R}}\;\right\}$ and ${\hat{S}}_{n}=\left\{\;\left(i,S_{n,i}\right)\;|\;i\in I_{N_{R}}\;\right\}$ are adjacent iff $S_{m,i},\;S_{n,i}$ are adjacent, for all $i\in I_{N_{R}}$, whereas two compound cells ${\hat{C}}_{i}^{}$ and ${\hat{C}}_{j}^{}$ are adjacent iff ${\hat{C}}_{i}^{\left[k\right]}$ and ${\hat{C}}_{j}^{\left[k\right]}$ are adjacent, for all $k\in I_{N_{R}}$. A path Π of configuration space cells is defined as any finite string of sequentially adjacent compound cells. Obviously, a path Π consisting of cells that lie entirely in $C^{f}$ and contain both $P_{\mathrm{init}}$ and $P_{\mathrm{des}}$ is a valid solution to our path finding sub-problem. In order to discover such a path, we build a hierarchical decomposition $H=\left\{\;\left(i,H\right)\;|\;i\in I_{N_{R}}\;\right\}$ of the configuration space C by assigning to each robot *i* a hierarchical partitioning of the workspace W, represented as a connected, directed tree $H≜\left(N_{H},E_{H}\right)$ such that:

- Each node $S\in N_{H}$ is a simple slice.
- Every child $S_{j}$ of a given node $S_{i}$ (i.e., $\left(S_{i},S_{j}\right)\in E_{H}$) is a strict subset of $S_{i}$.
- The set of leaf nodes must form a cover of W.

Finally, an algorithm was devised for appropriately expanding H until a solution is found, as described in the following subsection.

### 3.2. High-Level Planner

In this subsection, we present a high-level planner for finding a sequence Π of adjacent cells in $C^{f}$ connecting the initial $P_{\mathrm{init}}$ and goal $P_{\mathrm{des}}$ configurations. One of the main advantages of the proposed algorithm is the use of a suitable labeling scheme, which allows it to recursively subdivide, at each iteration, configuration space cells that lie on the boundary between $C^{f}$ and $C^{o}$ while ignoring cells that lie completely inside $C^{f}$ or $C^{o}$. To do so, this labeling scheme exploits the over- and under-approximations $\bar{R_{i}}$ and $\underline{R_{i}}$ of each robot’s footprint, defined in Section 3.1, to determine whether a robot may collide with another one while each robot navigates independently within its respective workspace cell. More specifically, given a compound cell ${\hat{C}}_{}^{}$, the employed cell labeling scheme works as follows:

- If the intersection of all $\bar{R_{i}}\left({\hat{C}}^{\left[i\right]}\right),\;i\in I_{N_{R}}$ is empty, then, by virtue of (4), no robot may come across another while $P\in {\hat{C}}_{}^{}$; thus, ${\hat{C}}_{}^{}$ is entirely contained in $C^{f}$. Such a compound cell is marked as *admissible*.
- If the intersection of all $\underline{R_{i}}\left({\hat{C}}^{\left[i\right]}\right),\;i\in I_{N_{R}}$ is non-empty, then, by virtue of (4), for every $P\in {\hat{C}}_{}^{}$ there exists at least one pair of intersecting robots; thus, ${\hat{C}}_{}^{}$ is entirely contained in $C^{o}$. Such a compound cell is marked as *inadmissible*.
- If ${\hat{C}}_{}^{}$ is neither admissible nor inadmissible, it is marked as *mixed*.

In general, mixed cells encapsulate both feasible and infeasible configurations and expanding them (recursively) should yield admissible and inadmissible subcells. On the other hand, by virtue of (5), the subdivision of admissible (resp., inadmissible) cells yields only admissible (resp., inadmissible) cells, without contributing any further in the configuration space’s exploration.

The planner’s main search algorithm is described in Algorithm 1, which initially constructs a coarse compound slice hierarchical partitioning made of each robot’s feasible set $A_{i}\left(W\right)$, thus enclosing all $C^{f}$ (functions InitializeHierarchy and InitializeCCells). Then, the initially computed compound cells get expanded until admissible ones, containing $P_{\mathrm{init}}$ and $P_{\mathrm{des}}$, are found (function FindEnclosingACCell), whereas the inability to find such compound cells indicates infeasibility of the given problem and the algorithm terminates. Next, an initial path Π connecting ${\hat{C}}_{\mathrm{init}}$ or ${\hat{C}}_{\mathrm{goal}}$ made of compound cells belonging to the exploration’s frontier set $S_{\hat{C},F}$ (i.e., the set of unexpanded admissible and mixed cells) is built (function ConnectStrings). At each iteration, the first mixed compound cell of Π (function GetFirstMixedCCell) is removed from the frontier and is expanded (function ExpandCCell) by subdividing the widest simple cell C whose over-approximation $\bar{R_{i}}\left(C\right)$ intersects with another (functions GetConflictingSCells and SelectSCellWithWidestSSlice) into smaller ones, as seen in Algorithm 2. Finally, a new path is constructed using standard back-tracking techniques until either Π consists only of admissible cells or no new path of mixed and admissible cells leading to $P_{\mathrm{des}}$ can be found.
**Algorithm 1** Planner’s Main Algorithm**function**FindAPath( $P_{\mathrm{init}}$, $P_{\mathrm{des}}$ )    H← InitializeHierarchy    $S_{\hat{C}}\leftarrow$InitializeCCells(H)    $S_{\hat{C},F}\leftarrow S_{\hat{C}}$    ${\hat{C}}_{\mathrm{init}}$, H, $S_{\hat{C}}$, $S_{\hat{C},F}$ ← FindEnclosingACCell( $P_{\mathrm{init}}$, H, $S_{\hat{C}}$, $S_{\hat{C},F}$)    ${\hat{C}}_{\mathrm{goal}}$, H, $S_{\hat{C}}$, $S_{\hat{C},F}$ ← FindEnclosingACCell( $P_{\mathrm{des}}$, H, $S_{\hat{C}}$, $S_{\hat{C},F}$)    **if** ${\hat{C}}_{\mathrm{init}}$ is null or ${\hat{C}}_{\mathrm{goal}}$ is null **then**        **return** null    **end if**    Π← ConnectStrings( $\left[{\hat{C}}_{\mathrm{init}}\right]$, $\left[{\hat{C}}_{\mathrm{goal}}\right]$, $S_{\hat{C},F}$ )    **while** not ((Π is null) or IsAdmissibleP(Π)) **do**        Π, H, $S_{\hat{C}}$, $S_{\hat{C},F}$ ← ExpandPath( Π, H, $S_{\hat{C}}$, $S_{\hat{C},F}$ )    **end while**    **return** Π**end function****function**FindEnclosingACCell( *P*, H, $S_{\hat{C}}$, $S_{\hat{C},F}$ )Given a point *P* in the configuration space of the robotic system, this function identifies the cell in the hierarchy H that contains that point and, if $S_{\hat{C}}$ is mixed, it iteratively subdivides $S_{\hat{C}}$ (modifying the hierarchy H in the process) until it obtains a subcell $S_{\hat{C},F}$ of $S_{\hat{C}}$ that contains *P* and is admissible.    $\hat{C}\leftarrow$FindCCellContainingPos(*P*, $S_{\hat{C},F}$)    **while** $\hat{C}$ is not admissible) **do**        i,S,C← SelectSCellWithWidestSSlice($\hat{C}$)        $S_{{\hat{C}}_{R}}$, H, $S_{\hat{C}}$, $S_{\hat{C},F}$ ←         ExpandCCell( $\hat{C}$, *i*, S, C, H, $S_{\hat{C}}$, $S_{\hat{C},F}$ )        $\hat{C}\leftarrow$FindCCellContainingPos(*P*, $S_{\hat{C},F}$)    **end while**    **return** $\hat{C}$, H, $S_{\hat{C}}$, $S_{\hat{C},F}$**end function****function**ConnectStrings( $\left[{\hat{C}}_{\mathrm{init}}\right]$, $\left[{\hat{C}}_{\mathrm{goal}}\right]$, $S_{\hat{C},F}$ )This function corresponds to some standard graph search algorithm (e.g., depth-first, breadth-first, A*) which, given: (a) the initial $\left[{\hat{C}}_{\mathrm{init}}\right]$ and desired $\left[{\hat{C}}_{\mathrm{goal}}\right]$ compound cells (in case two paths of cells are passed as arguments, the last cell of the first path and the first cell of the second path are used as $\left[{\hat{C}}_{\mathrm{init}}\right]$ and $\left[{\hat{C}}_{\mathrm{goal}}\right]$, respectively), (b) a set of compound cells (nodes) $S_{\hat{C},F}$ and their adjacencies (edges), yields either a sequence of admissible and mixed cells connecting $\left[{\hat{C}}_{\mathrm{init}}\right]$ and $\left[{\hat{C}}_{\mathrm{goal}}\right]$ if such a path exists or null otherwise.**end function**

**Algorithm 2** Path Expansion at Mixed Compound Cell
**function**ExpandPath( Π, H, $S_{\hat{C}}$, $S_{\hat{C},F}$ )    $\hat{C}\leftarrow$GetFirstMixedCCell(Π)    $L_{\mathrm{pre}}$, $L_{\mathrm{suf}}$ ← SplitString(Π, $\hat{C}$)    $S_{C}\leftarrow$ GetConflictingSCells($\hat{C}$)    i,S,C← SelectSCellWithWidestSSlice($S_{C}$)    $S_{{\hat{C}}_{R}}$, H, $S_{\hat{C}}$, $S_{\hat{C},F}$ ← ExpandCCell( $\hat{C}$, *i*, S, C, H, $S_{\hat{C}}$, $S_{\hat{C},F}$ )    Π← ConnectStrings$\left(L_{\mathrm{pref}},L_{\mathrm{suf}},S_{\hat{C},F}\right)$    **return** Π, H, $S_{\hat{C}}$, $S_{\hat{C},F}$
**end function**
**function**SplitString(Π, $\hat{C}$)Given a path Π and a specified cell $\hat{C}$ in Π, return two subsequences $L_{\mathrm{pre}}$ and $L_{\mathrm{suf}}$ of Π such that Π = ($L_{\mathrm{pre}}$, $\hat{C}$, $L_{\mathrm{suf}}$).
**end function**
**function**SelectSCellWithWidestSSlice($S_{C}$)Given a set of simple cells $S_{C}$, return the simple cell in $S_{C}$, the bounding box of which has the largest length or width.
**end function**



### 3.3. Velocity Control Law

**Figure 4:**
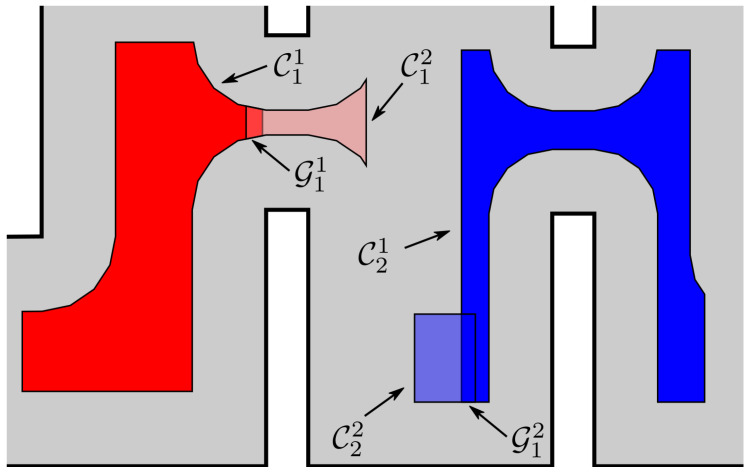
Two adjacent compound cells ${\hat{C}}_{1}=\left(C_{1}^{1},C_{1}^{2}\right)$ and ${\hat{C}}_{2}=\left(C_{2}^{1},C_{2}^{2}\right)$. In order for robot 1 (resp., robot 2) to successfully move from $C_{1}^{1}$ to $C_{2}^{1}$ (resp., from $C_{1}^{2}$ to $C_{2}^{2}$), it has to reach any point of $G_{1,1}$ (resp., $G_{2,1}$).

Given now a path Π consisting of $N_{\Pi }$ admissible configuration space cells, we present a distributed control law for safely navigating from one cell to the next until the goal configuration $P_{\mathrm{des}}$ is reached. First, we consider two consecutive compound cells ${\hat{C}}_{\ell }^{}$ and ${\hat{C}}_{\ell +1}^{}$ in Π, for which we compute the goal set $G_{i,\ell }≜{\hat{C}}_{\ell }^{\left[i\right]}\cap {\hat{C}}_{\ell +1}^{\left[i\right]}$ of each robot *i*, which contains feasible configurations in both ${\hat{C}}_{\ell }^{\left[i\right]}$ and ${\hat{C}}_{\ell +1}^{\left[i\right]}$ and is non-empty by construction. Respectively, the goal set corresponding to the last cell of Π consists of just the desired configuration $P_{\mathrm{des}}$, i.e., ${\hat{C}}_{N_{\Pi }}^{}=\left\{P_{\mathrm{des}}\right\}$ (see Figure 4). Furthermore, let $F_{\ell ,i}≜{\hat{C}}_{\ell }^{\left[i\right]}\setminus \mathrm{int}\left(G_{\ell ,i}\right)$ and $G_{\ell ,i}^{\prime }≜G_{\ell ,i}\cap F_{\ell ,i}$, for all $k\in I_{N_{\Pi }}$. Notice that $G_{\ell ,i}^{\prime }$ is generally made of one or more pairwise disjoint subsets of arbitrary connectedness as well as that robot *i* should navigate to any of these regions without escaping ${\hat{C}}_{\ell }^{\left[i\right]}$ in order to successfully traverse to the next specified workspace cell. When more than one of such disjoint goal subsets are reachable from the connected component of $F_{\ell ,i}$ that contains $p_{i}$, one of them is arbitrarily (though, deterministically) selected and assigned as the goal set; a more sophisticated approach for choosing goal regions could be employed, but it exceeds the scope of the current work. Respectively, the transition from ${\hat{C}}_{\ell }^{}$ to ${\hat{C}}_{\ell +1}^{}$ is considered complete after every robot *i* reaches ${\hat{C}}_{\ell +1}^{\left[i\right]}$. We also remark that when ${\hat{C}}_{\ell }^{\left[i\right]}\subseteq {\hat{C}}_{\ell +1}^{\left[i\right]}$, robot *i* simply needs to retain its current position during step *ℓ*.

In order to fulfill the aforementioned specifications, we equip each robot *i* with a controller $u_{i}$ based on suitable workspace transformations and adaptive artificial potential fields, which were originally presented in [27] and possess guaranteed domain invariance and almost-global convergence properties. More specifically, we build a diffeomorphic transformation $q_{i}^{\ell }=T_{i}^{\ell }\left(p_{i}\right)$ that maps $F_{\ell ,i}$ to the unit disk D, the outer boundary of $F_{\ell ,i}$ to the unit circle ∂D and collapses all inner boundaries to distinct points $q_{i,j}^{\ell },\;j\in I_{N_{i}^{\ell }}$, where $N_{i}^{\ell }$ is the genus of $F_{\ell ,i}$. We now distinguish the following two cases of possible goal sets: (a) $G_{\ell ,i}^{\prime }$ being an inner boundary of $F_{\ell ,i}$, and (b) $G_{\ell ,i}^{\prime }$ being part of the outer boundary of $F_{\ell ,i}$. Depending on the case, $T_{i}^{\ell }$ must be appropriately adapted to simplify the subsequent potential field’s design. Particularly, case (a) can be accommodated by modifying $T_{i}^{\ell }$ such that $G_{\ell ,i}$ collapses to an inner point $q_{\mathrm{des},i}^{\ell }$ of D, whereas case (b) is addressed by designing $T_{i}^{\ell }$ such that $G_{\ell ,i}^{\prime }$ collapses to a single point $q_{\mathrm{des},i}^{\ell }$ on ∂D. Next, we define the harmonic potential field $\phi_{i}^{\ell }$ used by robot *i* during step *ℓ* by placing point harmonic sources upon the corresponding goal configuration $q_{\mathrm{des},i}^{\ell }$ and the transformed inner obstacles $q_{i,j}^{\ell }$, given by:

(7) $$\phi_{i}^{\ell }=k_{i,d}^{\ell }\ln\left(\frac{∥q_{i}^{\ell }-q_{\mathrm{des},i}^{\ell }∥}{2}\right)-\sum_{j\in I_{N_{i}^{\ell }}}k_{i,j}^{\ell }\ln\left(\frac{∥q_{i}^{\ell }-q_{i,j}^{\ell }∥}{2}\right)$$

where $k_{i,d}^{\ell }>0$ and $k_{i,j}^{\ell }\geq 0$ are adaptively varying parameters. Finally, the control law $u_{i}^{\ell }$ of robot *i* during step *ℓ* is given by

(8) $$u_{i}^{\ell }=-Ks\left(q_{i}^{\ell },k_{v,i}^{\ell }\right){\left(J_{i}^{\ell }\left(q_{i}^{\ell }\right)\right)}^{-1}\nabla_{q_{i}^{\ell }}\psi_{i}^{\ell }\left(q_{i}^{\ell },k_{v,i}^{\ell }\right)$$

where *K* is a positive control gain, $J_{i}^{\ell }$ is the Jacobian matrix of $T_{i}^{\ell }$, *s* is a factor ensuring collision avoidance with the outer boundary and $\psi_{i}^{\ell }=1+\tanh(w\phi_{i}^{\ell })/2$, with *w* a positive constant.

## 4. Simulation Results

**Figure 5:**
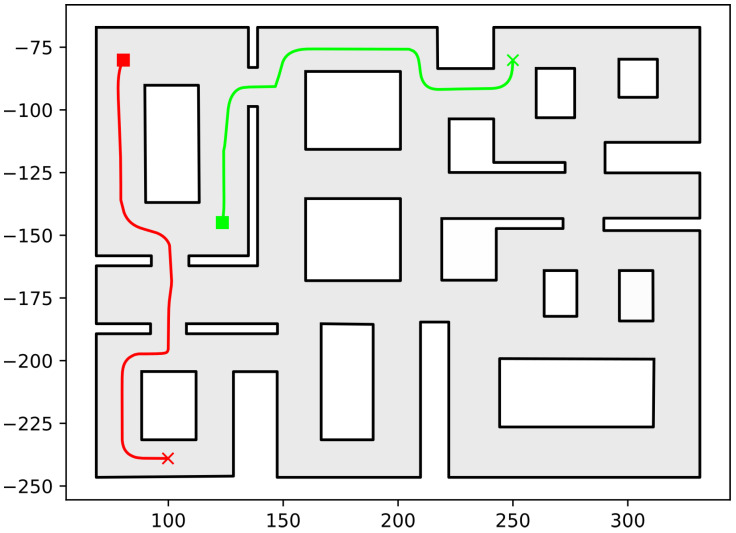
Executed trajectories of the two-robot case. Squares indicate initial positions, whereas the corresponding goal positions are depicted using crosses. Robot #1 (red); Robot #2 (green). The axes units are in (dm).

**Figure 6:**
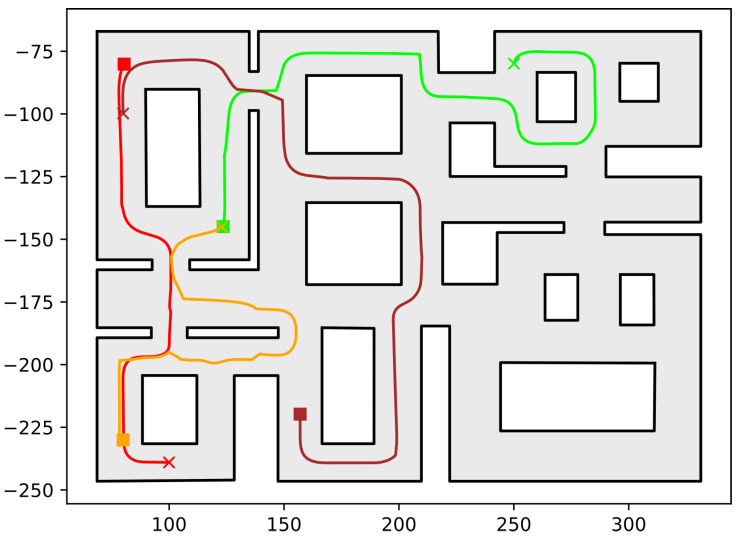
Executed trajectories of the four-robot case. Squares indicate initial positions, whereas the corresponding goal positions are depicted using crosses. Robot #1 (red); Robot #2 (green); Robot #3 (dark red); Robot #4 (orange). The axes units are in (dm).

**Figure 7:**
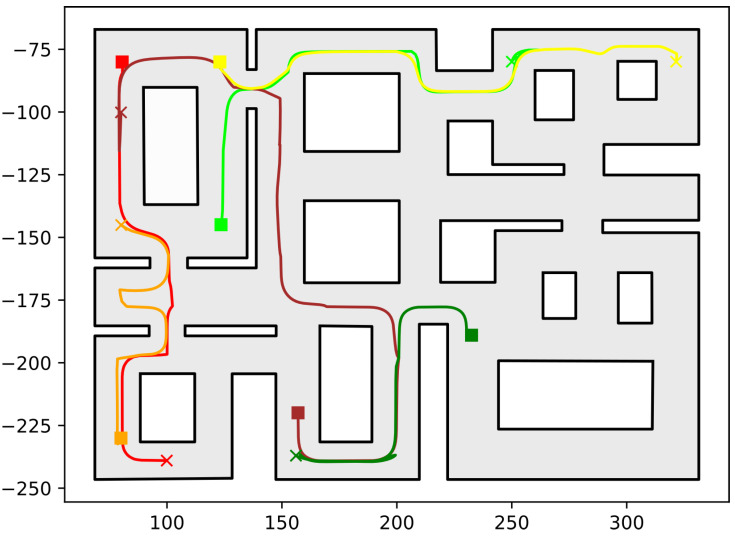
Executed trajectories of the six-robot case. Squares indicate initial positions, whereas the corresponding goal positions are depicted using crosses. Robot #1 (red); Robot #2 (green); Robot #3 (dark red); Robot #4 (orange); Robot #5 (yellow); Robot #6 (dark green). The axes units are in (dm).

**Figure 8:**
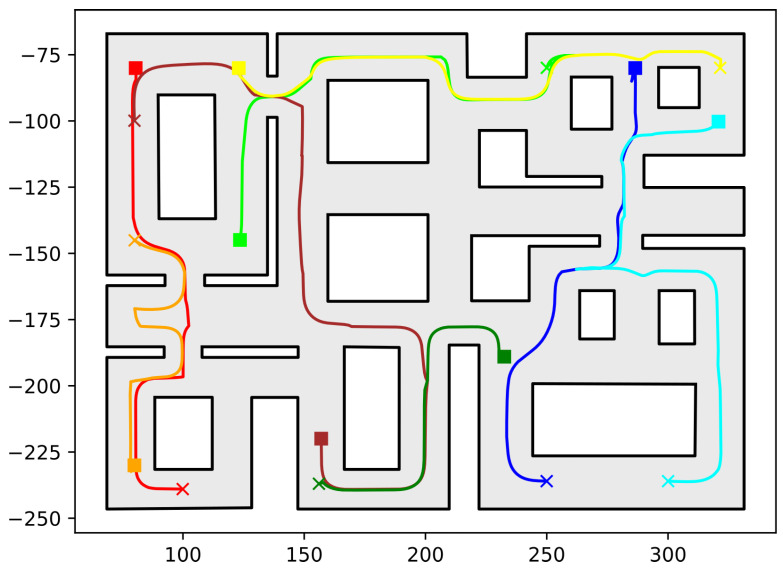
Executed trajectories of the eight-robot case. Squares indicate initial positions, whereas the corresponding goal positions are depicted using crosses. Robot #1 (red); Robot #2 (green); Robot #3 (dark red); Robot #4 (orange); Robot #5 (yellow); Robot #6 (dark green); Robot #7 (blue); Robot #8 (cyan). The axes units are in (dm).

**Figure 9:**
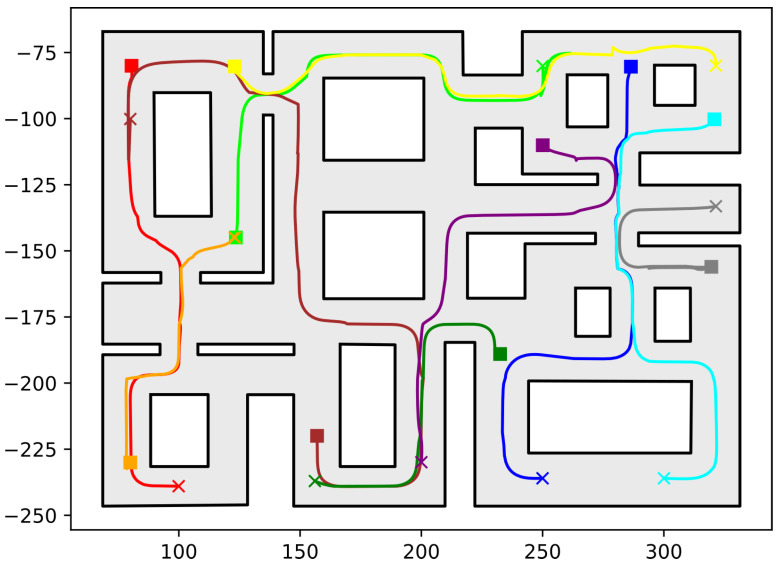
Executed trajectories of the ten-robot case. Squares indicate initial positions, whereas the corresponding goal positions are depicted using crosses. Robot #1 (red); Robot #2 (green); Robot #3 (dark red); Robot #4 (orange); Robot #5 (yellow); Robot #6 (dark green); Robot #7 (blue); Robot #8 (cyan); Robot #9 (purple); Robot #10 (grey). The axes units are in (dm).

**Figure 10:**
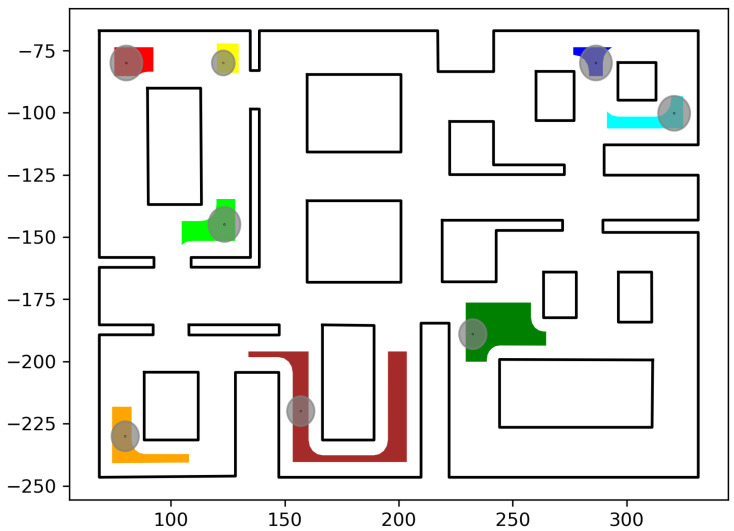
The initial robot positions $p_{\mathrm{init},i},\;i\in I_{8}$ and calculated initial compound cell ${\hat{C}}_{\mathrm{init}}$. Robot #1 (red); Robot #2 (green); Robot #3 (dark red); Robot #4 (orange); Robot #5 (yellow); Robot #6 (dark green); Robot #7 (blue); Robot #8 (cyan). The axes units are in (dm).

**Figure 11:**
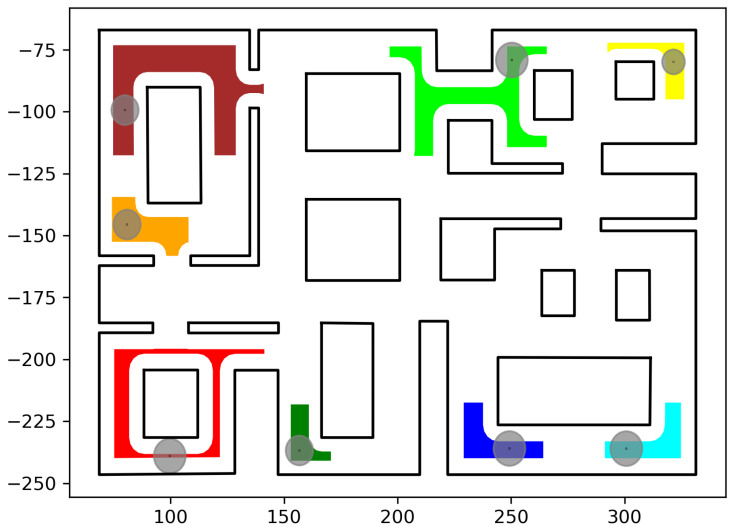
The desired robot positions $p_{\mathrm{des},i},\;i\in I_{8}$ and calculated goal compound cell ${\hat{C}}_{\mathrm{goal}}$. Robot #1 (red); Robot #2 (green); Robot #3 (dark red); Robot #4 (orange); Robot #5 (yellow); Robot #6 (dark green); Robot #7 (blue); Robot #8 (cyan). The axes units are in (dm).

In this section, we present the simulation results demonstrating the efficacy of the proposed methodology. As the contribution of this work is a path-planning algorithm, simulations were deemed sufficient to validate the efficacy of the proposed method. To conduct these simulations, the algorithm described in this work was implemented in C++, using Boost Geometry and CGAL for performing the geometric operations, such as the configuration computation, subdivision of cells, etc. The implementation can be found at the following url: https://github.com/maxchaos/mrnav, accessed on 20 December 2022. As described, the configuration space was partitioned into a tree of cells and a standard graph search algorithm was employed for finding admissible paths of mixed cells connecting the initial and goal configurations. After such a path was obtained, for each compound cell in the sequence, we extracted the simple cells corresponding to each robot, and for each robot, we employed the low-level control scheme to drive it to the border of the next cell or its goal configuration in case the current cell was the last of the path. Particularly, we consider five scenarios where a system consisting of 2, 4, 6, 8 and 10 robots, respectively, initialized within the workspace depicted in Figure 5 is requested to reach a specified final configuration. The time required by the proposed planner, as well as the total amount of compound cells generated during the solution of each case, are shown in Table 1. We remark that the planner expanded the mixed compound cells by subdividing the corresponding conflicting simple slice into four identical overlapping sub-slices. The motion profiles executed by the robots in each corresponding case can be seen in Figure 5, Figure 6, Figure 7, Figure 8 and Figure 9. Additionally, Figure 10 and Figure 11 depict the initial and goal configurations as well as the computed enclosing cells, respectively, for the eight-robot scenario. As one can verify from the figures, the robots navigate successfully to their individual goals. A video of the aforementioned simulated scenarios can be found at the following url: https://youtu.be/asWnKLNX2Eg, accessed on 20 December 2022.

To strengthen our theoretical findings, we also conducted a comparative simulation study over the same scenarios employing a state-of-the-art high-level planner called RRT* [4] that is based on a probabilistic sampling algorithm to extract feasible paths toward the goal configuration. Because RRT* is probabilistic, we ran 50 trials on each scenario and extracted the mean value of the execution time and total path as described in Table 2. We have to stress that owing to the narrow passages of the workspace, as the number of robots increased (particularly for the cases of 8 and 10 robots), the RRT* algorithm reached the maximum terminating condition (an execution time more than 1000 s) very frequently (many runs did not discover feasible paths) without providing feasible solutions. Notice that our algorithm is superior in terms of the execution time and completeness without however sacrificing the performance in terms of the total path length. It should be pointed out that although our algorithm does not incorporate any optimization in either the path calculation or the transition execution, the achieved performance is comparable to the RRT* algorithm, which finds the shortest path among multiple feasible ones.

## 5. Conclusions

In this work, we presented a hybrid control scheme for addressing the navigation problem of a team of robots operating within an obstacle-cluttered planar workspace. Particularly, a high-level planner was designed for computing a sequence of feasible cells by adaptively subdividing the system’s configuration space using a hierarchical cell decomposition scheme. In addition, a low-level control law based on harmonic potentials and workspace transformations was employed to implement the given plan, safely navigating each robot to its specified goal. The contribution lies on the adaptive subdivision algorithm for obtaining a sequence of traversable cells connecting the given initial and final configurations or determining that no solution exists. Unlike standard grid/cell-based methods, the proposed algorithm adaptively subdivides the cells, requiring no selection of some arbitrary resolution by the user. Moreover, unlike probabilistic sampling methods, the proposed method does not generate paths of configuration but rather sequences of safe sets, allowing the user to make use of a larger set of controllers in addition to standard trajectory tracking schemes. In addition, unlike probabilistic sampling methods which are inherently incapable of determining the infeasibility of a given problem (such algorithms require the user to specify an arbitrary upper bound of samples after which the algorithms abort) as a consequence of their probabilistic completeness nature and the fact that sample points should lie within the configuration space, our algorithm partitions the configuration space into the cells (dense subsets) of the configuration space and is capable of determining when no admissible path exists in finite time. Finally, comparative simulation studies were conducted for various scenarios with a state-of-the-art algorithm that validates the proposed scheme’s efficacy and demonstrates its superiority in terms of complexity while yielding a satisfactory performance in terms of path lengths.

Regarding the limitations of the proposed approach and future research directions toward healing them, we aim to improve the proposed planner by accommodating for redundant steps appearing in the computed path and adapt it for use in a more distributed fashion, i.e., resolve the conflicts between antagonistically operating robots as they appear. Additionally, introducing optimality in the selection of the sequence of cells as well as in the execution of the transitions deserves further investigation to improve the efficiency of the proposed method. In the same vein, increasing the dimensionality of the problem (considering 3D workspaces and potentially the orientation) would also favor its applicability. Alternatively, a modern approach such as deep reinforcement learning [28] will be sought to examine new ways of solving the multi-robot coordination problem.

## Figures and Tables

**Table 1:** Execution time, total length of the robots’ paths and number of generated compound cells required by the high-level planner for solving each scenario.

**Number of Robots**	**2**	**4**	**6**	**8**	**10**
Time (sec)	0.088	0.240	0.845	1.36	31.1
Length (dm)	404.94	1076.23	1396.20	1739.53	2125.36
Compound Cells	51	348	823	1014	3363

**Table 2:** Average and standard deviation for the execution time and total length of the robots’ paths over the number of successful runs yielded by the RRT* planner [4] for solving each scenario over 50 runs.

**Number of Robots**	**2**	**4**	**6**	**8**	**10**
Time-avg (sec)	0.041	0.124	12.375	724.136	961.230
Time-std (sec)	0.001	0.045	0.454	30.032	41.211
Length-avg (dm)	380.15	1112.21	1256.82	1712.45	1995.63
Length-std (dm)	1.25	5.18	7.32	15.82	17.31
Successful runs	50	50	49	36	5

## Data Availability

Not applicable.
